# A resonance frequency analysis to investigate the impact of implant size on primary and secondary stability

**DOI:** 10.12669/pjms.40.6.8213

**Published:** 2024-07

**Authors:** Mahvish Wahad Khan, Naveed Inayat, Muhammad Sohail Zafar, Abdul Mueed Zaigham

**Affiliations:** 1 Mahvish Wahad Khan, BDS, FCPS Assistant Professor, Department of Prosthodontics Avicenna Medical & Dental College, Phase IX, DHA, Bedian Road, Lahore, Pakistan; 2 Naveed Inayat, BDS, FCPS, FICD Associate Professor, Department of Prosthodontics Azra Naheed Dental College, Superior University, Lahore, Pakistan; 3 Prof. Muhammad Sohail Zafar, BDS, M.Sc, PhD Department of Restorative Dentistry, College of Dentistry, Taibah University, Al Madina, Al Munawwarra 41311, Saudi Arabia. Department of Dental Materials, Islamic International Dental College, Riphah International University, Islamabad 44000, Pakistan.; 4 Prof. Abdul Mueed Zaigham, BDS, FCPS Department of Prosthodontics, Institute of Dentistry, CMH Lahore Medical College, Abdur Rehman Road, Lahore. National University of Medical Sciences, Rawalpindi, Pakistan

**Keywords:** Implant stability, Primary stability, Secondary stability, Resonance frequency, Implant stability quotient (ISQ)

## Abstract

**Objective:**

Recent years have seen a rise in the usage of dental implants to restore lost teeth. The stability of a dental implant is the main factor in determining its success. Implant stability is influenced by various factors. Several approaches have been employed clinically to evaluate stability at different time intervals. One non-invasive way to assess implant stability is by resonance frequency analysis. Utilizing the resonance frequency analysis method, this study seeks to understand how implant length and diameter affect primary and secondary stability.

**Methods:**

The current prospective study was conducted in the Prosthodontics Department of Institute of Dentistry, CMH Lahore Medical College. The duration of the study was six months. A total of 90 implants of sizes 4.5 x 8.5 mm and 4 x 10mm were placed. Resonance frequency measurements were recorded using Osstell™ AB device for primary stability at implant insertion and at 12 weeks for secondary stability. All the measurements were carried out by only one of the researchers to minimize inter-observer bias.

**Results:**

The average primary stability was 70.33±6.60, and the average secondary stability was 71.43±5.44. The data was stratified for age, gender, and implant site, and the mean primary and secondary stability of both sizes didn’t show any statistically significant differences.

**Conclusion:**

Without forfeiting implant stability, both implant sizes (4 x 10mm and 4.5 x 8.5mm) can be used interchangeably, depending on available space and anatomical constraints.

## INTRODUCTION

In recent years, replacing missing teeth with fixed or removable dental prosthesis supported by dental implants has gained popularity.^1^ Dental implants have emerged as a predictable treatment modality to accomplish good functional and aesthetic outcomes.^2^ They are surgically inserted into the jawbone and should be in direct contact with bone under ideal circumstances, which is referred to as osseointegration.^3^

The fundamental element that determines whether a dental implant will succeed is, its stability. The absence of clinical movement demonstrates an implant’s stability. This property of a dental implant can only be achieved by combining osseous tissue with alloplastic material without intervening fibrous connective tissues.^4^ Primary stability and secondary stability are the two categories of stability. The lack of movement in bone just after implant placement is primary stability.^5^ Secondary stability is an improvement in stability caused by peri-implant bone growth, which includes new bone formation at the implant-bone interface along with progressive bone remodeling and osteoconduction.^6^ It requires approximately 12 weeks for the transition from primary to secondary stability to occur.

Primary stability is mechanical^7^ which is achieved by compression stress generated in the bone, influenced by implant design/geometry, length, diameter, local bone characteristics, and surgical technique whereas secondary stability is biological in nature,^8^ attributable to osseointegration.^9^ Primary stability, bone remodeling, and implant surface conditions are all factors that influence secondary stability.^10^ Implant stability plays a critical role for successful osseointegration, which is a pre-requisite for functional dental implants. Implant mobility owing to lack of osseointegration results in implant failure. Short implants cause implant failure due to unfavorable crown root ratio whereas implants with a smaller diameter are less likely to withstand stresses leading to implant components fracture.^11^

Various invasive and non-invasive methods have been employed to evaluate implant stability at different time intervals. Resonance frequency analysis (RFA) is thought to be the only approach that can objectively and non-invasively measure implant stability and osseointegration development without endangering the healing process in intra and post-operative settings.^4^,^12^ The Osstell device utilizes RFA for the measurement of implant stiffness and mobility on a scale having range of 1-100 to determine implant stability quotient (ISQ).^5^,^13^ ISQ gives information about the axial perspective of stability.^14^

The primary ISQ varies with different implant diameters (3.75 and 4.25mm), but not with varied implant lengths (10mm and 11.5mm). Although diameter had an impact on the secondary ISQ, no other parameters had a significant impact.^9^ Longer implants are more stable ones, even when the bone is of low quality.^5^ This study evaluated the impact of length and diameter on dental implant’s primary and secondary stability so that in the future implants of appropriate length and diameter could be selected with predictable stability values and failures could be avoided.

## METHODS

This study was carried out in the Prosthodontic Department, Institute of Dentistry, CMH Lahore Medical College Lahore.

The duration of the study was six months. A sample size of 90 patients was calculated at 95% confidence level and 1% margin of error and taking the expected primary ISQ value for diameter 4.25 as 77.04±4.24.^6^ Non-probability consecutive sampling technique was adopted. Systemically healthy individuals of both genders with ages between 18-60 years (ASA I/II) requiring implants to replace missing teeth after six months of extraction were included in the study. Patients on oral bisphosphonates and those with current major systemic health issues or oral pathologies (assessed on medical records and clinical examination) were excluded. After taking informed written consent, implants of diameters 4.5 x 8.5 mm and 4 x 10 mm were placed at various sites in partially edentulous patients. The implant size was selected according to the residual bone width and height along with consideration of sufficient distance from vital anatomic structures.

### Ethical Approval:

It was obtained from the Ethical Review Board of the college (105/ERC/CMH/LMC). Date: July 10, 2018.

**Fig.1:**
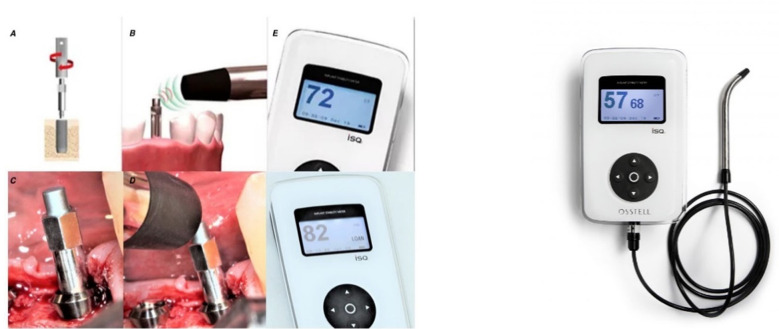
OstellTM ISQ Device. A. Smart peg & Smart Peg-Mount. B,C,D. Performing a measurement E. Instrument Display.

The implant length and diameter were noted for each patient and every site. Osstell ISQ device (Osstell AB, Stampgatan, and SE-411 01 Goteborg, Sweden. Fig.1) was used to measure ISQ at the time of implant insertion and after 12 weeks. This gadget utilizes RFA technology, which is founded on the tuning fork principle. It performs a bending test by stimulating a transducer to apply a minuscule bending force. It is comparable to delivering a constant lateral push to an implant and monitoring the implant’s displacement. The measurement unit is Implant Stability Quotient (ISQ) which is a linear mapping from resonance frequency measured in kHz to the more clinically useful scale of 1-100 ISQ.^8^

The ISQ values were noted respectively for primary and secondary stability. All the measurements were carried out by the researcher herself to minimize inter-observer bias. Demographic variables (age, gender), implant site and outcome variables were recorded on a pre-designed performa. With the aid of a transporter implant fixture was fastened with an Osstell® Smart Peg threaded transducer. The Smart Peg automatically transformed resonance frequencies into ISQ values. The median of two measurements along the buccolingual and mesiodistal axes served as the basis for all values.

### Statistical Analysis:

SPSS version 22 was used to enter the data. Mean and Standard deviation were calculated for quantitative variables like age, primary and secondary ISQ value. For gender and implant site, frequency and percentages were computed. The Chi-square test was used to determine the p-value. A p-value of ≤ 0.05 was considered significant. To compare the primary and secondary ISQ values, a T-test was performed. Data was stratified according to implant site, gender, and age. Chi-square and t tests were utilized post-stratification.

## RESULTS

The study comprised of 90 individuals who came to the department for missing tooth replacement. The average age of patients was 36.62±9.08 years. There were 52 men (57.8%) and 38 women (42.2%). There was a male-to-female ratio of 1.4:1. The most frequent site receiving dental implants was the posterior mandible where 41 implants were placed followed by the posterior maxilla which received 34 implants. The most frequently placed size was 4 x 10 mm. In total 47 implants of this size were placed (Chart-1). While the difference between their mean values was statistically insignificant, the primary stability value ranged from 55 to 79, and the secondary stability value ranged from 59 to 81, suggesting that implant stability increases over time. The mean insertion torque was 33.72±2.55 (Table-I). The mean primary and secondary stability for both implant sizes were calculated respectively, and the shift was not statistically significant (p > 0.05). The mean primary stability among candidates with an implant size of 4.5x8.5mm was 70.12±6.40. 70.53±6.64.84 was the mean primary stability among candidates whose implant size was 4x10mm. The mean secondary stability in candidates with an implant size of 4.5x8.5mm was 71.12±5.44. 71.72±5.48 was the mean secondary stability in candidates with implant size 4x10mm (Table-II). Data was stratified for implant site for both implant sizes for primary as well as secondary stability and the p-value was found to be insignificant, (Table-III).

**Table-I:** The mean primary and secondary stability and insertion torque were calculated. ISQ and IT of patients.

		Primary stability	Secondary stability	Insertion torque (IT) (mm)
ISQ	N	90.00	90.00	90.00
	Mean	70.33	71.43	33.72
	SD	6.60	5.44	2.55
	Minimum	55.00	59.00	30.00
	Maximum	79.00	81.00	40.00

**Table-II:** Primary and secondary stability comparison in both implant sizes.

	Implant size (mm)	4.5x 8.5	4 x 10		Implant size (mm)	4.5 x 8.5	4 x 10
Primary stability (ISQ)	N	43.00	47.00	Secondary stability (ISQ)	N	43.00	47.00
	Mean	70.12	70.53		Mean	71.12	71.72
	SD	6.40	6.84		SD	5.44	5.48
	Minimum	58.00	55.00		Minimum	60.00	59.00
	Maximum	79.00	79.00		Maximum	80.00	81.00
P-value	0.767 (Insignificant)				0.600 (Insignificant)		
t-test value	0.297				0.526		

**Table-III:** Comparison of primary and secondary stability in both implant sizes stratified for the implant site.

	Implant Site		Anterior Maxilla			Anterior Mandible			Posterior Maxilla				Posterior Mandible			

			N	Mean	SD	n	Mean		SD		N	Mean	SD	N	Mean	SD
**Primary stability**	Implant size mm	4.5x8.5	0.00	0.00	0.00	0.00	0.00		0.00		18.00	68.06	6.45	25	71.60	6.06
		4x10	9.00	71.11	6.88	6.00	73.33		2.16		16.00	68.88	7.24	16.00	70.81	7.62
	P-value		NA			NA					0.729			0.716		

			*N*	*Mean*	*SD*	*N*		*Mean*		*SD*	*N*	*Mean*	*SD*	*N*	*Mean*	*SD*

**Secondary stability**	Implant size mm	4.5x8.5	0.00	0.00	0.00	0.00		0.00		0.00	18.00	69.89	5.97	25.00	72.00	4.97
		4x10	9.00	71.00	5.32	6.00		74.67		4.32	16.00	72.13	6.69	16.00	70.63	4.60
	P-value		NA			NA					0.311			0.379		


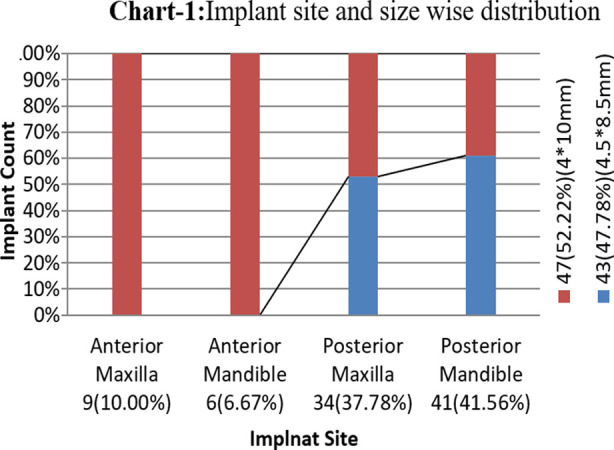


## DISCUSSION

The success of dental implants undoubtedly depends upon their stability.^15^ The most critical clinical goal to achieve at the time of implant insertion is primary stability, which dictates secondary stability as well.^8^ RFA has greater potential to predict implant stability while being non-invasive and reproducible^14^, which is why it was chosen as the method to measure stability values in the present study.

Poor primary implant stability impedes osseointegration; causing the formation of fibrous tissue at the implant-bone interface.^12^ The RFA technique is simply a bending test in which a transducer is stimulated to apply an exceedingly tiny bending force. In terms of direction and kind, it is analogous to delivering a fixed lateral force to the implant and measuring its displacement. This effectively simulates clinical loading circumstances, albeit on a much smaller scale. At any stage of therapy, the RFA approach has the ability to offer clinically useful information on the state of the implant-bone interface.^16^

In the current study, implant sizes 4.5 x 8.5mm and 4mm x 10mm were selected because they were the most frequent sizes observed to have been used previously for tooth replacements in the Prosthodontics Department. Resonance frequency measurements were recorded with the Osstell™ AB device at the time of implant insertion and at 12 weeks. All measurements were carried out by only one of the researchers to minimize inter-observer bias. Newer methods for measuring stability values have also been introduced and should be considered for use in future research.

The mean primary stability in candidates with implant size 4.5 x 8.5mm was 70.12±6.40 and in candidates with implant size 4x10mm it was 70.53±6.84. The mean secondary stability was 71.12±5.44 in candidates with implant size 4.5x8.5mm and 71.72±5.48 in candidates with implant size 4x10mm. It has been found that the stability of dental implants enhanced during the shift from primary to secondary stability. Previous research also proved that good primary/mechanical stability leads to more efficient secondary/biological stability accomplishment.^17^ Another study also reported that from insertion to osseointegration, secondary stability increases as a result of bone growth.^9^ Literature also suggests implant stability only increases during the healing process for implants with poor initial stabilities. Implants with strong initial stabilities can experience stability loss while healing.^18^

The current study demonstrated insignificant difference in the implant stability with the increase in diameter (from 4 to 4.5mm) or with the change in length (8.5 -10mm). In another study it was also reported that the narrow diameter and standard diameter implants show comparable initial stability, which supports the findings of the current study.^19^ In yet another study no significant differences were reported in narrow, regular, and wide diameter implants success after 1-year and 3-year follow-ups.^20^

One more study used 4.1mm & 4.8mm diameter implants and observed that ISQ values at baseline and at 4,8, and 12 weeks for both diameters did not differ.^21^ It can therefore be inferred that implant diameter does not significantly affect the stability values. According to Sabeva’s research, increasing implant diameter has a greater positive impact on primary stability than increasing implant length. But it should be remembered that this corresponds to a precise modification of 1.5 mm in diameter and 2mm in length.^22^ To support or refute this relationship, additional research is required, including implants with a bigger length differential and a different diameter-to-length ratio. The insignificant findings of our study could be due to the fact that only a difference of 0.5mm in diameter and 1.5mm in length was there in the selected implant sizes. Gomez-Polo et al. reported that the primary ISQ varies depending on the patient’s bone quality and implant diameter (3.75 and 4.25) but did not differ between the two lengths (10mm and 11.5mm) compared. According to the findings of his research, none of these characteristics had a significant impact on secondary stability.^9^ These findings were in accordance with the results of the current study, except regarding primary stability, which was reported to be significantly better with larger diameter implants by the researcher however this was not true as per the findings of our study.

Our investigation found that increasing the length of an implant did not result in an increase in primary stability. Contrarily, Bataineh and Al-Dakes found that increasing dental implant length is expected to play a significant role in enhancing primary stability, even in low-quality bone, by managing the bone preparation process.^5^ Barikani et al. in his study used three implant lengths small, medium, and long (10, 13, 16mm) and three diameters: narrow platform, regular platform, and wide platform (3.5, 4.3, and 5mm). He concluded that an increase in length from medium to long results in higher stability. 13mm long implants of all diameters had appropriate primary stability regardless of other factors. The primary stability of the wide platform (WP) (5) was not different from the regular platform (RP) (4.3) as less bone was removed.^23^

Interestingly in a study by Aragoneses JM et al. a direct relationship between implants of a smaller length and greater ISQ values was reported, with this relationship being most evident in the maxilla and in women.^24^ Contrary to this no statistically significant correlation was found between width and length on ISQ value at placement or follow-up in a study where implants of dimensions 4.3 × 10, 4.3 × 13, and 5 × 10 mm were placed in relatively equal amounts.^25^ The above findings endorse the result of our study.

Current study showed, no increase in primary stability was seen with the increase in diameter because the implant diameters used in this study were 4 & 4.5mm which do not fall under the category of small diameters. Although it has been suggested that increasing implant diameter could improve the bone-implant connection area and decrease the movement of implant at the time of insertion.^26^ The use of implants with smaller diameters (4 mm) is another significant problem; one study found that employing implants with smaller diameters resulted in decreased implant stability and survival rates. Additionally, the smaller diameter of the implant may cause relatively significant levels of bone stress.^27^

No significant difference was noted in mechanical as well as biological stability with the increase in diameter as per the investigations of current study. However, Nappo et al., reported that the mean primary stability (ISQ) was significantly lower (71.38±5.79) with 3.5 mm implant as compared to 4mm implant (76.23±5.16, p<0.05), while secondary stability (ISQ) was also significantly lower (78.46±6.43) with 3.5 mm implant as compared to 4mm implant (79.89±5.73, p<0.5).^28^

Implant size, shape and surface characteristics are included in implant macrogeometry, all of which play a critical role in its primary stability. However, 12 mm marks the end of the linear association between implant length and primary stability.^29^ Using long and wide implants, can be potentially challenging requiring a more invasive surgical approach and difficulty in accurate placement.

The primary stability of the dental implants in this study varied depending on the location, with the anterior mandible having the highest level of stability, followed by the anterior maxilla and posterior mandible, and the posterior maxillary region having the lowest level of stability. Intraoral location was correlated with ISQ values in another study and implants placed in the mandible were found to be more stable than those placed in the maxilla.^25^ Monje et al. also reported higher implant stability in mandible compared with maxilla immediately after insertion and 4 months later.^30^ In another prior study, it was also suggested that there was a connection between bone density at the receptor site and implant primary stability.^31^

Mechanical stability as well as biological stability with larger diameter implants was more in the posterior mandible whereas with longer implants it was more in the anterior mandible. Mean primary/mechanical stability as well as mean secondary/biological stability was least in the posterior maxilla for both implant sizes. While the majority of studies link higher bone densities to more stable implants, some studies reported the contrary. These discrepancies in results are most likely caused by the various methodologies used in these investigations. For instance, different researchers have used different methods to determine bone quality. The term “bone quality” is inadequately explained in academic studies. Additionally, the earlier researches used various techniques to gauge initial stability.^18^

### Limitations

he limitation of the current study was the use of only two implant sizes with slight differences in their lengths and diameters (the difference between implant diameters was 0.5mm while the difference in length was 1.5mm) which could be the reason for insignificant findings in relation to how length and diameter affect the stability values. Further studies should be conducted using a variety of implant sizes so that a more significant inference could be made about the role of implant diameter and length on its success and long-term survival.

## CONCLUSION

Implant size (length and diameter) is part of the broader term “implant macrogeometry”. Increasing implant length and diameter increases functional surface area and primary stability, which in turn increases secondary stability. No significant difference was observed in the primary/mechanical as well as secondary/biological stability between the implant sizes used in this study. Both implant sizes are equally effective in their respective places and can be used interchangeably according to available space without compromising the stability of the implants.

### Authors’ Contribution:

**MWK:** Conceptualization, methodology, investigation, data curation, original draft preparation and editing of manuscript. Responsible and accountable for the accuracy and integrity of work.

**NI:** Draft preparation, literature search, software application, data analysis.

**MSZ:** Visualization, critical review, supervision, references management.

**AMZ:** Proof reading, project administration and supervision.
